# Time to antenatal care booking and its predictors among pregnant women in East Africa: a Weibull gamma shared frailty model using a recent demographic and health survey

**DOI:** 10.3389/fgwh.2024.1457350

**Published:** 2024-11-27

**Authors:** Abel Endawkie, Shimels Derso Kebede, Kaleab Mesfin Abera, Eyob Tilahun Abeje, Ermias Bekele Enyew, Chala Daba, Lakew Asmare, Fekade Demeke Bayou, Mastewal Arefaynie, Anissa Mohammed, Abiyu Abadi Tareke, Awoke Keleb, Natnael Kebede, Yawkal Tsega

**Affiliations:** ^1^ Department of Epidemiology and Biostatistics, School of Public Health, College of Medicine and Health Sciences, Wollo University, Dessie, Ethiopia; ^2^ Department of Health Informatics, School of Public Health, College of Medicine and Health Sciences, Wollo University, Dessie, Ethiopia; ^3^ Department of Health System and Management, School of Public Health, College of Medicine and Health Sciences, Wollo University, Dessie, Ethiopia; ^4^ Department of Environmental Health College of Medicine and Health Sciences, Wollo University, Dessie, Ethiopia; ^5^ Department of Reproductive and Family Health, School of Public Health, College of Medicine and Health Sciences, Wollo University, Dessie, Ethiopia; ^6^ Amref Health Africa, West Gondar Zonal Health Department, Gondar, Ethiopia; ^7^ Department of Health Promotion, School of Public Health College of Medicine Health Sciences, Wollo University, Dessie, Ethiopia

**Keywords:** time to ANC booking, predictors, pregnant women, East Africa, Weibull gamma shared frailty modeling

## Abstract

**Background:**

Antenatal care (ANC) is an important component of maternal and child healthcare. The World Health Organization (WHO) recommends that pregnant women book their ANC contact at or before 12 weeks of gestational age. However, in East Africa, evidence on whether the WHO recommendations have been followed is limited. Therefore, this study aimed to determine the time to ANC booking and its predictors among pregnant women in East Africa.

**Method:**

This study was conducted among 86,662 pregnant women in East Africa. The time to ANC booking was estimated using the Kaplan–Meier (K–M) survival estimate. A Weibull gamma shared frailty model was used to determine the predictors of time to the first ANC visit. An adjusted hazard ratio (AHR) with a 95% confidence interval (CI) was reported.

**Result:**

The median time to ANC booking among pregnant women in East Africa was 4 ± 2 months. Maternal education at the primary (AHR = 1.01, 95% CI: 1.02–1.25), secondary (AHR = 1.03, 95% CI: 1.02–1.05), and higher level (AHR = 1.40, 95% CI: 1.30–1.50); husband's education level at the primary (AHR = 1.08, 95% CI: 1.06–1.09), secondary (AHR = 1.12, 95% CI: 1.10–1.13), and higher (AHR = 1.08, 95% CI: 1.07–1.10) levels as compared to with no education; a middle-class wealth status (AHR = 1.66, 95% CI: 1.60–1.70), being rich (AHR: 1.60, 95% CI: 1.56–1.73), high community-level maternal literacy (AHR = 1.05, 95% CI: 1.04–1.06), high community-level poverty (AHR = 0.99, 95% CI: 0.98–0.99), previous Cesarean section (CS) (AHR = 1.35, 95% CI: 1.33–1.39), and unwanted pregnancy (AHR = 0.74, 95% CI: 0.72–0.77) were predictors of the time to ANC booking.

**Conclusion:**

The median time to ANC booking among pregnant women in East Africa is longer than the new WHO recommendation. Maternal and husband education, high community-level maternal literacy, a better household, community-level wealth index, and previous CS increase the likelihood of an early ANC booking. However, unwanted pregnancy lowers the likelihood of an early ANC booking. Therefore, strengthening systematic efforts to improve women’s and their husbands' educational status, encouraging women's education in the community, providing economic support for women with low wealth status and poor communities, encouraging wanted pregnancy, and providing accessible counseling services for women with unwanted pregnancies will help to encourage early ANC booking among pregnant women in East Africa.

## Introduction

Antenatal care (ANC) plays a crucial role in maternal and child health by focusing on enhancing the wellbeing of pregnant women and their developing babies (1). Antenatal care involves a series of medical tests and interventions that are aimed at ensuring the health and safety of both the pregnant woman and the developing fetus throughout the pregnancy (2).

The World Health Organization (WHO) that recommends pregnant women have an ANC booking within the first 12 weeks of gestation to reduce perinatal mortality and improve women's experience of care (3). Early antenatal care refers to a woman initiating prenatal care before or at 12 weeks of gestation, ensuring timely monitoring and intervention for a healthy pregnancy (3).

Approximately 295,000 girls died globally from preventable causes related to pregnancy and childbirth, an average of 810 deaths per day (4). Most of these deaths (94%) occurred in areas where resources were limited and many could have been prevented (4). Sub-Saharan Africa (SSA) accounted for more than two-thirds (196,000) of all maternal deaths globally with a large burden in East Africa (4). The global maternal mortality rate (MMR), which refers to maternal deaths per 100,000 live births, is approximately 216, with a massive 95% in developing countries (5).

Despite the recent launch of Sustainable Development Goals (SDGs) Target 3.1 that aimed to reduce MMR to 70 per 100,000 live births globally by 2030 (6), MMR remains a significant public health challenge in SSA (7). Delayed initiation of ANC visits may result in adverse maternal and neonatal outcomes, including maternal morbidity and mortality, preterm delivery, low birth weight, and regional neonatal mortality (4, 5).

Despite the increasing availability of antenatal care in many African countries, coverage alone does not adequately measure service utilization and the time at which they initiate the first time of ANC visit is a concern (8).

According to different studies, the factors related to time to ANC booking were the education status of women, marital status of the woman, husband's occupation and level of education, media exposure, household wealth index, community women's literacy, the interval between births, transportation issues, and distance to the health facility (9–14).

Although there is extensive evidence of the prevalence and associated factors for ANC booking among reproductive-age women (10, 15–22) using categorical data analysis, evidence related to time to ANC booking and its predictors among pregnant women using the time-to-event model (10, 19, 23) in different countries of East Africa is limited. Moreover, there is a paucity of evidence for a time to ANC booking that follows the WHO recommendation using recent Demographic and Health Survey (DHS) data from 2016 to 2023 in East Africa (3). This study provides important insights into the barriers and facilitators of timely ANC booking in East Africa, which can inform evidence-based interventions to improve maternal and child health outcomes in the region. Therefore, this study aimed to determine the time to ANC booking and its predictors among pregnant women in East Africa using recent DHS data following the 2016 WHO recommendation for ANC booking for a positive pregnancy experience.

## Method

### Study design and settings

The DHS used a cross-sectional study design to collect data on various health, nutrition, and demographic indicators and this study was conducted based on recent DHS data among pregnant women in East Africa from 2016 to 2023. Ethiopia, Tanzania, Rwanda, Uganda, Kenya, Comoros, and Burundi were included in this study.

### Inclusion and exclusion

The countries that have recent DHS datasets following the 2016 WHO recommendation of ANC visits for a positive pregnancy experience were selected (3). However, countries with no DHS data such as Somalia, Eritrea, South Sudan, Sudan, and Djibouti were excluded from the analysis.

### Source and study population

The source populations of this study were all pregnant women before the survey in East Africa, whereas those in the selected enumeration areas (EAs) were the study population.

#### Data source

We used recent the birth record (BR) dataset in the DHS after authorization was granted via an online request on the DHS program's official website (https://dhsprogram) and extracted the dependent and independent variables. The DHS is a nationally representative household survey that is conducted across low and middle-income countries every 5 years. It has been an essential data source for issues in maternal healthcare utilization in low- and middle-income countries as it gathers data on several reproductive health issues such as marriage, ANC visits, postnatal visits, fertility preferences, and contraception. The data collected from the DHS survey are organized in a hierarchical structure, with households within a cluster forming the top level. The next level consists of household members, followed by interviewed women and men as a subset of household members. The bottom levels of the hierarchy include the pregnancies and children of each interviewed woman (24, 25).

#### Sample size and sampling method

The study included a total of 86,662 pregnant women from the recent round surveys in seven East African countries. The study participants were selected using a two-stage stratified sampling technique. EAs were randomly selected in the first stage, while households were randomly selected in the second stage.

#### Study variables

The dependent variable of this study is the time to ANC booking (in months) among pregnant women from 0 to 9/10 months of pregnancy.

The independent variables included sociodemographic and economically related factors such as maternal age, maternal educational status, marital status, religion, husband's educational status, sex of the household head, age of the household head, household wealth index, residence, community-level wealth index, community-level maternal literacy, country, birth interval, types of pregnancy, and previous Cesarean section (CS).

### Measurement and operational definitions

#### Time

The time taken in months from the beginning of a woman’s pregnancy to an ANC booking during the indexed pregnancy.

#### Event

Whether the mother made at least one ANC booking during the indexed pregnancy.

#### Censored

Whether the mother had at least one ANC booking during the indexed pregnancy.

#### Community-level poverty

The proportion of women originating from households classified within the poorest and poor wealth index categories was summed and then divided by the total household wealth index value of each cluster. The households with a wealth index value at or above the mean were classified as being at a high poverty level, while those below the mean were categorized as having a low poverty level. The mean was chosen as the cutoff point in this context due to the normal distribution observed in the poverty level at the community level, as indicated by the coefficient of skewness falling between −1 and 1, signifying a normal distribution.

#### Community-level maternal literacy

The proportion of mothers who were in the primary and above categories was summed and then divided by the total maternal educational status value of each cluster. The mothers of a child with a maternal educational status value at or above the mean were classified as experiencing a high level of maternal literacy, while those below the mean were categorized as having a low level of maternal literacy. The mean was chosen as the cutoff point in this context due to the normal distribution.

### Data processing and analysis

The data were extracted, cleaned, coded, and analyzed using STATA version 17 statistical software. Sample weights were applied before further analysis, and descriptive statistics were summarized using frequencies, percentages, medians, and interquartile ranges, which are presented in tables, figures, and narrative form. The time to ANC booking was estimated using the Kaplan–Meier (K–M) survival estimate. The log-rank test was used to check the statistical significance of the variable in the bivariable Cox regression analysis. Variables that had a *p*-value <0.25 in the bivariable analysis were selected for multivariable analysis in both models (Cox and Weibull gamma). After the proportional hazard (PH) assumptions for Cox regression and stratified Cox regression were tested and failed, we conducted a parametric survival analysis of the frailty model to identify predictors of time to ANC booking among pregnant women in East African countries.

#### Shared frailty model

To determine predictors of time to ANC booking among pregnant women in East African countries, we utilized a shared frailty model due to the hierarchical nature of the data and its correlation at the cluster level. A random effect of the shared frailty model assumes that the frailties are common (or shared) among groups of individuals and are randomly distributed across groups. A shared frailty model has a latent multiplicative effect on the hazard function and is assumed to have a unit mean and variance of θ, which is estimated along with the other model parameters (26). We conducted a parametric survival analysis of different shared frailty models. The Weibull gamma shared frailty model was the best-fit model based on the Akaike information criterion (AIC) and Bayesian information criterion (BIC) for model selection. Finally, the adjusted hazard ratio (AHR) was reported at a 95% confidence interval (CI) and a *p*-value <0.05 indicated the statistical significance of predictors of the time of the ANC booking.

#### Ethical approval

No ethical approval was needed because we used the Demographic and Health Survey, which de-identifies all data before making it public. An authorization letter was required to download the DHS dataset which was then obtained from the Central Statistical Agency (CSA) after being requested at https://dhsprogram.com/. The dataset and all methods of this study were conducted according to the guidelines in the Declaration of Helsinki and were based on DHS research guidelines.

## Results

### Sociodemographic and health service-related characteristics of the study participants

The data from a total of 86,662 pregnant women were analyzed. The mean age of the women was 28 years with a standard deviation of 7 years. Among the pregnant women, 12,484 (98.3%) who made an ANC booking were married. Of the mothers with no education, 7,805 (98.4%) made an ANC booking. In addition, 23,089 (97.7%) of the women who had made an ANC booking were from urban areas.

In total, 24 (0.2%) women from Burundi, 121 (1.2%) from Rwanda, 476 (13.2%) from Ethiopia, and 825 (14.5%) from Tanzania did not make an ANC booking. Of the pregnancies, 73,893 (98.14%) were wanted by mothers who had made an ANC booking. Concerning previous obstetric history, 9,325 (99.5%) of mothers with a previous CS delivery made an ANC booking (Table 1).

**Table 1:** The sociodemographic and health service-related characteristics of the study participants with the time to ANC booking in East African countries using recent DHS data.

Variable	Category	Time to ANC booking			
		Censored		Event	
		Frequency	%	Frequency	%
Maternal age in groups (years)	15–19	205	4.8	4,088	95.2
	20–24	549	5.1	10,155	94.9
	25–29	6,365	26.1	18,059	73.9
	30–34	562	3.8	14,402	96.2
	35–39	527	4.9	10,241	95.1
	40–44	2,555	34.1	4,940	65.9
	45–49	1,234	8.8	12,780	91.2
Education	No education	861	5.97	13,570	94.03
	Primary	1,019	2.6	37,503	97.4
	Secondary	385	1.6	24,147	98.4
	Higher	15	0.2	9,163	99.8
Marital status	Union	223	1.7	12,484	98.3
	Not union	2,057	2.78	71,898	97.22
Religion	Christian	1,055	1.5	67,921	98.5
	Muslim	288	5.2	5,229	94.8
Sex of the household head	Male	1,957	2.9	65,283	97.1
	Female	323	1.7	19,099	98.3
Age of the household head (years)	15–21	302	14.2	1,830	85.8
	22–34	759	1.9	38,673	98.1
	35–64	1,145	2.7	40,315	97.2
	>64	74	2	3,564	98
Husband education status	No education	475	5.7	7,805	94.3
	Primary	683	2	32,916	98
	Secondary	439	2.4	17,494	97.5
	Higher	21	0.2	10,702	99.8
Number of household members	1–5	805	1.7	45,982	98.3
	6–7	513	2.2	22,687	97.8
	>8	963	5.8	15,712	94.2
Number of children under 5 years	1–4	2,266	2.6	84,088	97.4
	5	14	4.5	294	95.5
Household wealth index	Poor	1,496	4.5	31,520	95.5
	Middle	493	2.6	18,359	97.4
	Rich	291	0.8	34,503	99.2
Place of residence	Urban	537	2.3	23,089	97.7
	Rural	1,743	2.76	61,293	97.24
Country	Burundi	24	0.2	10,312	99.8
	Ethiopia	476	13.2	3,118	86.8
	Kenya	238	0.8	28,777	99.2
	Comoros	335	10.7	2,796	89.3
	Rwanda	121	1.2	9,861	98.8
	Tanzania	825	14.5	4,853	85.5
	Uganda	260	1.04	24,667	98.96
Preceding birth interval	Less than 2 years	556	5.6	9,429	94.4
	2 years	36	3.4	1,012	96.6
	Greater than 2 years	1,113	2.13	51,316	97.87
Previous CS delivery (*n* = 86,605)	No	2,239	2.9	75,000	97.1
	Yes	41	0.4	9,325	99.4
Type of pregnancy (*n* = 83,067)	Wanted	1,403	1.8	73,893	98.2
	Unwanted	400	5.14	7,371	94.86
Community-level maternal literacy	Low	2,053	4	51,866	96
	High	227	0.7	32,516	99.3
Community-level poverty	Low	878	2	43,577	98
	High	1,402	3.3	40,805	96.7

CS, cesarean section.

Study respondents for type of pregnancy (*n* = 83,067) and previous CS delivery (*n* = 86,605).

### Median time of ANC booking among pregnant women in East Africa

Out of the total 86,662 pregnant women surveyed, 84,382 mothers made an ANC booking (event), while 2,280 pregnant women did not make an ANC booking (censored) during the interview period. Among the 84,382 pregnant women who initiated ANC, 52,751 (62.54%) made the ANC booking within 12 weeks of gestation. The median time for the ANC booking was 4 months, with an interquartile range of 2 months (4 ± 2 months).

### Survival distribution and non-parametric survival analysis of the time to ANC booking among pregnant women in East Africa

**Figure 1:**
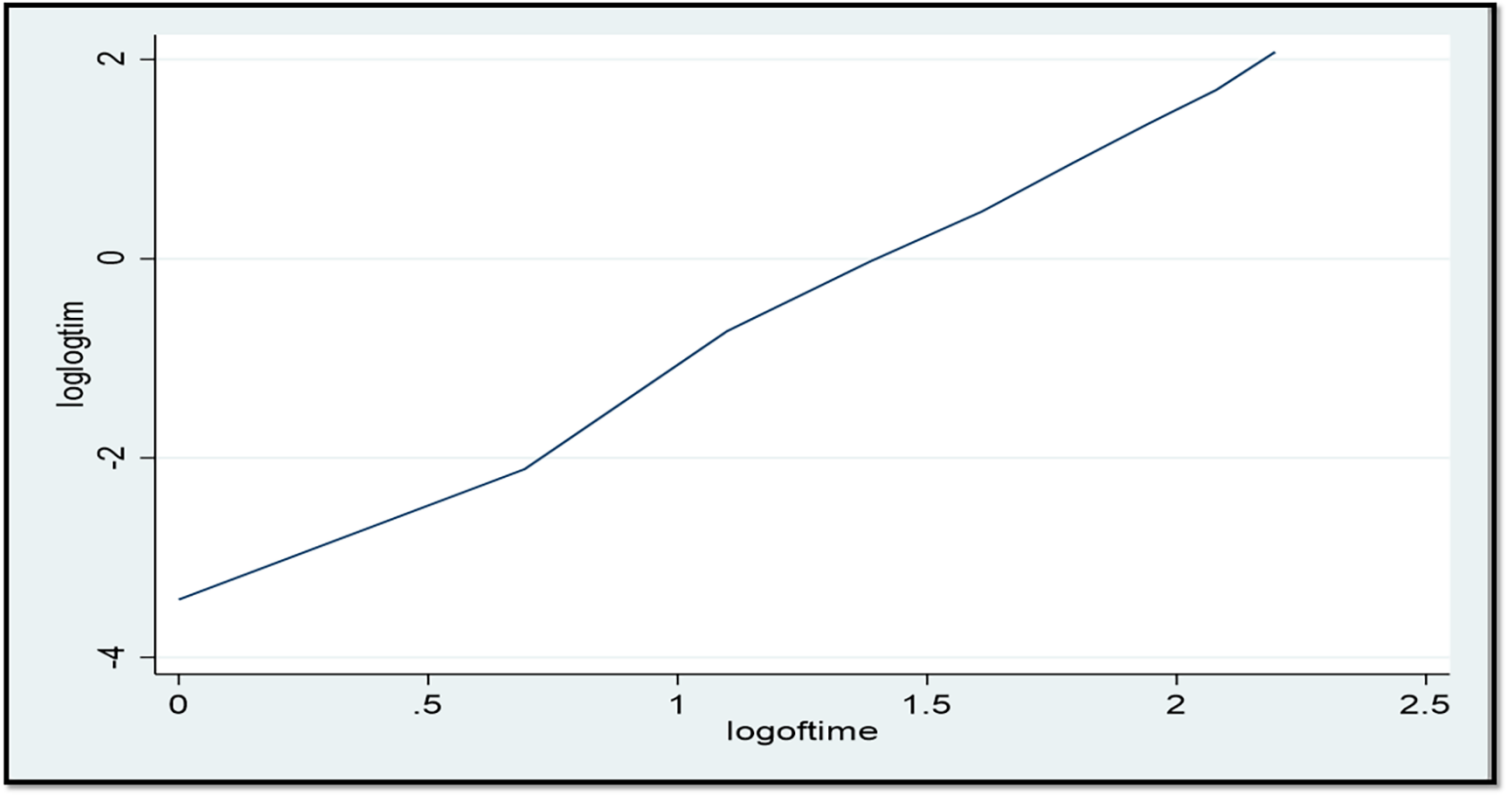
The Weibull distribution of the log-log of survival time vs. the log of time to first antenatal care visit in East Africa using recent DHS data.

**Figure 2:**
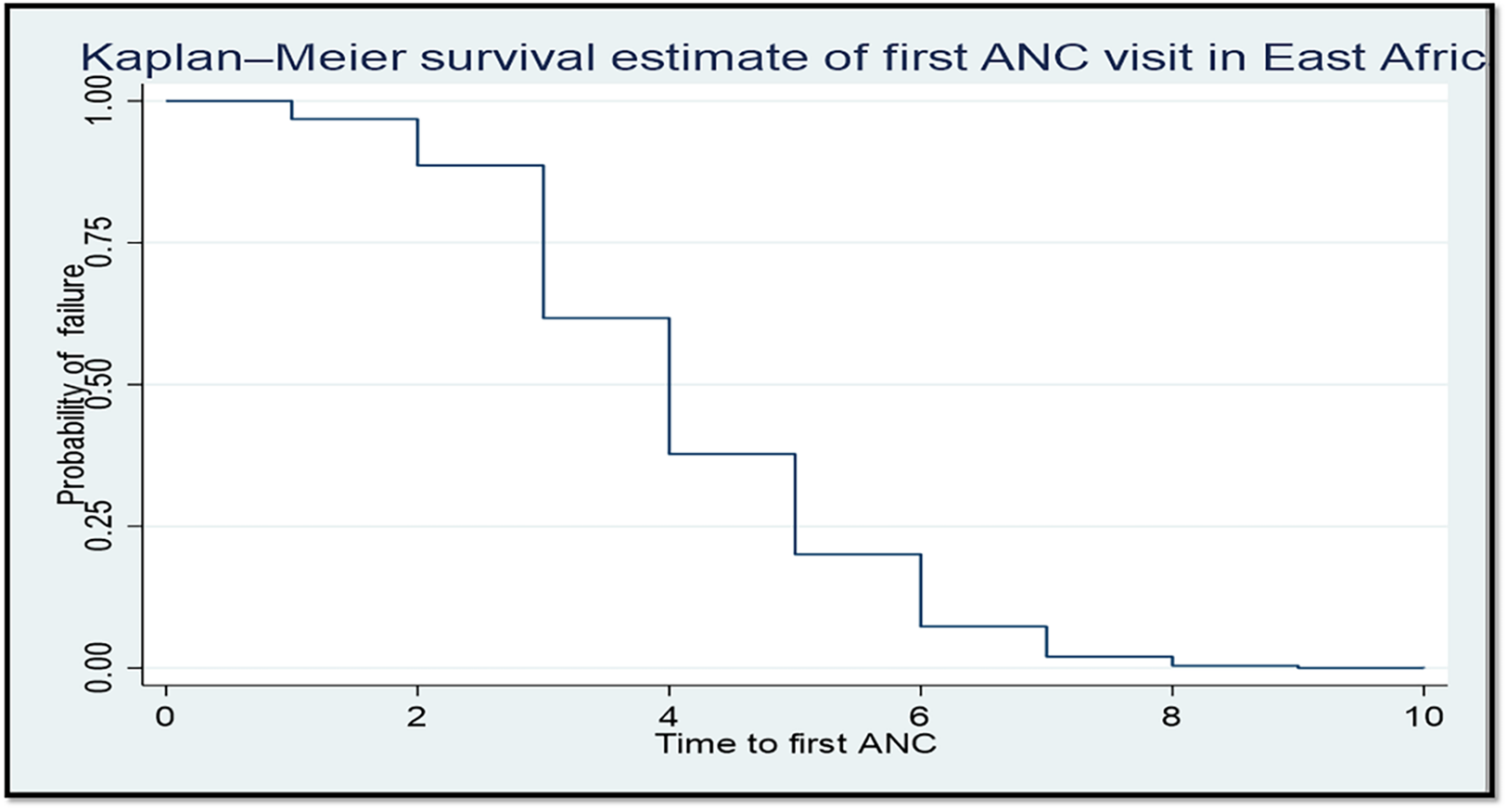
The Kaplan–Meier failure time of the first antenatal care visit among pregnant women in East Africa using recent DHS data.

**Figure 3:**
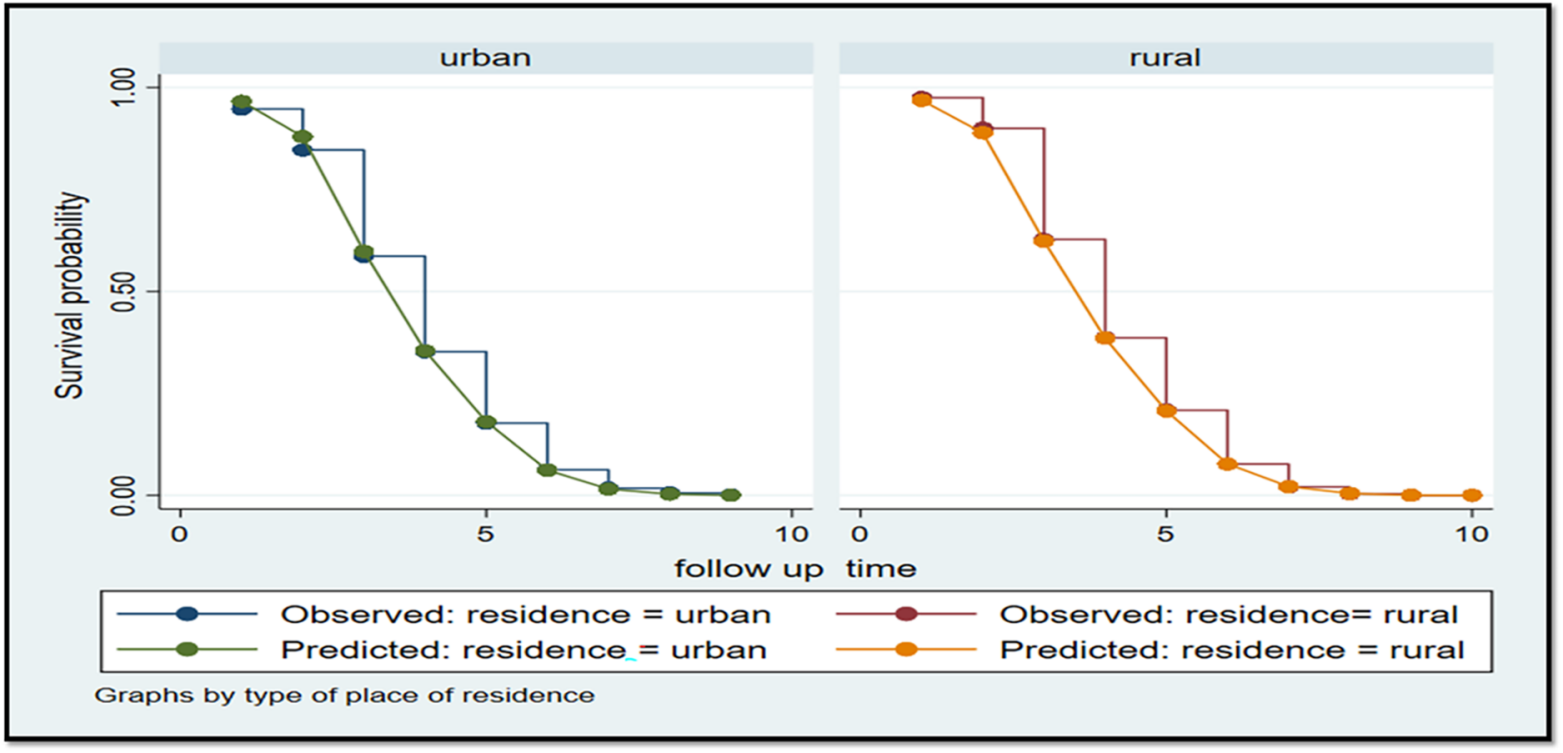
The Kaplan–Meier survival time of the first antenatal care visit based on pregnant women’s place of residence in East Africa using recent DHS data.

**Figure 4:**
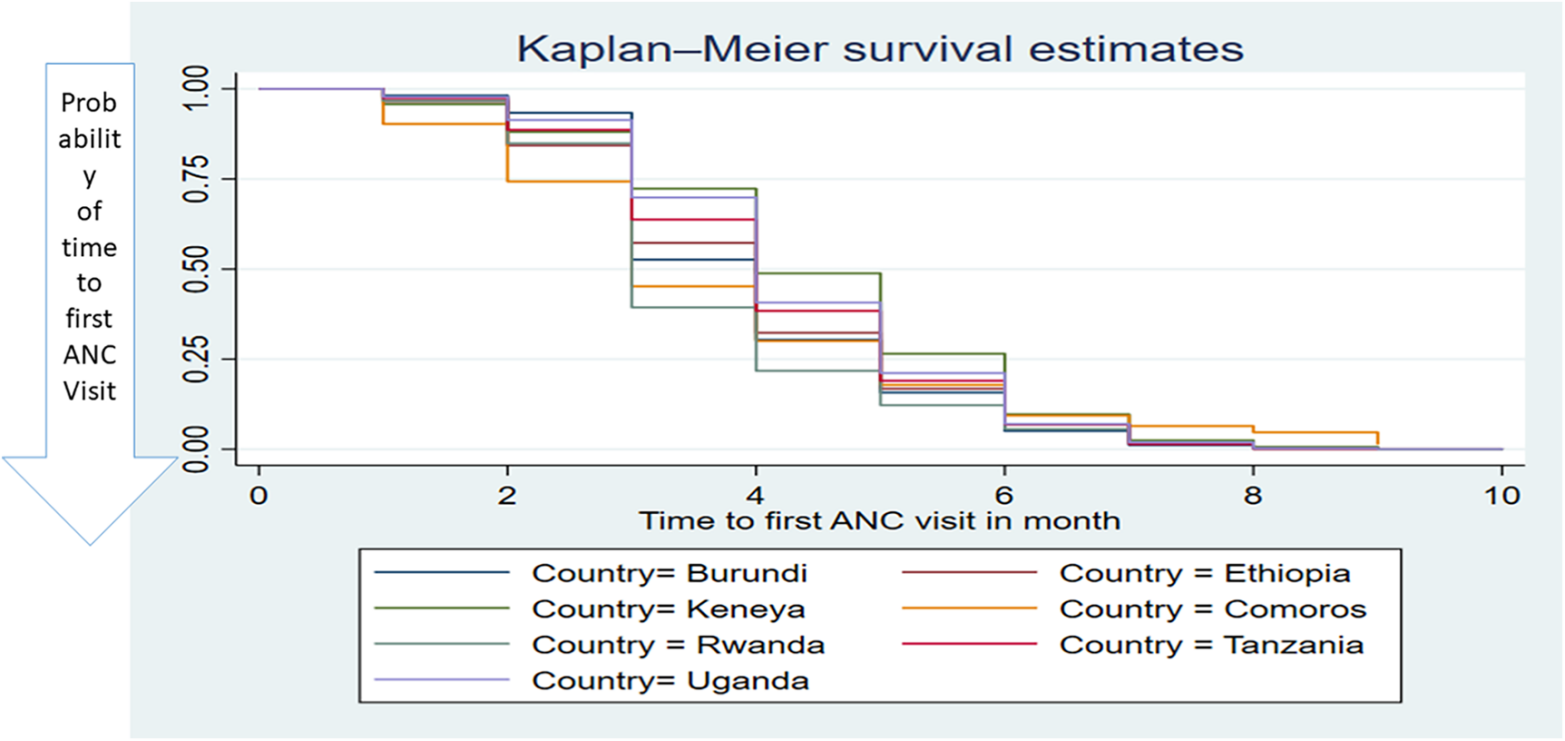
The Kaplan–Meier survival time of the first antenatal care visit among pregnant women based on countries in East Africa using recent DHS data.

A parametric survival model with a Weibull distribution was used to display the time to ANC booking among pregnant women in East Africa. The Weibull distribution compared the survival rates with the time of the ANC booking in East Africa using recent DHS data. The *x*-axis represents the log of time, while the *y*-axis represents the log-log of survival time, and as the time increases, the probability of making an ANC booking also increases (Figure 1). The Kaplan–Meier estimate of time to ANC booking is used to estimate the survival time of pregnant women in East Africa. The Kaplan–Meier estimates of time to ANC booking revealed that the probability of ANC booking was highest in the early gestational period and tended to decrease substantially with increasing gestational age (Figure 2). The Kaplan–Meier estimates of time to ANC booking among pregnant women in East Africa implied that the probability of ANC booking was higher in the early gestational period in urban residences compared to rural residences (Figure 3). The Kaplan–Meier estimates of time to ANC booking among pregnant women in East Africa imply that the probability of making an ANC booking was higher in the early gestational period in Burundi, Rwanda, and Uganda while it was higher in the later period in Tanzania and Ethiopia (Figure 4).

### Semi-parametric survival analysis (Cox regression analysis) to determine the predictors of time to ANC booking

The Schoenfeld residual test was conducted using the proportional hazard assumption to detect Cox regression model fitness and determine the predictors of time to ANC booking among pregnant women in East African countries. The global proportional hazard test was significant with a *p*-value = 0.000. To consider stratified Cox regression, we used the detailed proportional hazard test using the “estat phtest” command in Stata version 17. The detailed proportional hazard test showed that more than one variable was significant with a *p*-value <0.05 which made it difficult to conduct stratified Cox regression. Finally, we decided to conduct a parametric survival analysis to identify the predictors of time to ANC booking among pregnant women in East African countries.

### A Weibull gamma shared frailty model to determine the predictors of time to ANC booking among pregnant women in East Africa

To identify the predictors of time to ANC booking among pregnant women in East African countries, a shared frailty parametric survival analysis was conducted. Thus, a Weibull gamma shared frailty model for the parametric survival analysis was selected as it had the lowest AIC and BIC values (Table 2).

**Table 2:** Model comparison and selection for parametric survival analysis of shared frailty model.

Model	Model type	AIC	BIC
Model 1	Gompertz gamma frailty model	63,033.7	60,276.1
Model 2	Weibull inverse Gaussian frailty AFT model	59,907.7	60,176.1
Model 3	Weibull inverse Gaussian PH frailty model	59,987.6	60,135.1
Model 4	Weibull gamma frailty AFT model	59,896.2	60,376.1
Model 5	Weibull gamma frailty PH model	59,890.4	59,998.8

AFT, accelerated failure time; PH, proportional hazard; AIC, Akaike information criterion; BIC, Bayesian information criterion.

The predictors of time to ANC booking among pregnant women in East Africa were maternal age, maternal educational status, religious status of the woman, sex of the household head, age of household head, husband educational status, household size, household wealth index, community-level maternal literacy, community-level wealth index, previous CS delivery, and type of pregnancy (Table 3).

**Table 3:** The Weibull gamma shard frailty analysis of the predictors of time to ANC booking among pregnant women in East Africa using recent DHS data.

Variable	First ANC booking		95% CI of AHR
	Censored	Event	
Maternal age in groups (years)			
15–19	205	4,088	Reference
20–24	549	10,155	1.47 (1.40–1.53)*
25–29	6,365	18,059	1.40 (1.35–1.47)*
30–34	562	14,402	1.30 (1.20–1.90)*
35–39	527	10,241	1.30 (1.20–1.34)*
40–44	2,555	4,940	1.07 (1.06–1.09)*
45–49	1,234	12,780	0.92 (0.90–0.97)*
Education status of the woman			
No education	861	13,570	Reference
Primary	1,019	37,503	1.01 (1.01–1.03)*
Secondary	385	24,147	1.03 (1.02–1.05)*
Higher	15	9,163	1.40 (1.30–1.50)*
Religion			
Christian	1,055	67,921	Reference
Muslim	288	5,229	0.93 (0.92–0.94)*
Sex of the household head			
Male	1,957	65,283	Reference
Female	323	19,099	0.96 (0.95–0.97)*
Age of the household head (years)			
15–21	302	1,830	Reference
22–34	759	38,673	1.06 (1.03–1.08)*
35–64	1,145	40,315	1.09 (1.06–1.10)*
>64	74	3,564	1.06 (1.02–1.09)*
Husband education status			
No education	475	7,805	Reference
Primary	683	32,916	1.08 (1.06–1.09)*
Secondary	439	17,494	1.12 (1.10–1.13)*
Higher	21	10,702	1.08 (1.06–1.09)*
Number of household members			
1–5	805	45,982	Reference
6–7	513	22,687	1.02 (1.01–1.02)*
>8	963	15,712	1.07 (1.06–1.08)*
Household wealth index			
Poor	1,496	31,520	Reference
Middle	493	18,359	1.66 (1.60–1.70)*
Rich	291	34,503	1.60 (1.50–1.70)*
Place of residence			
Urban	537	23,089	Reference
Rural	1,743	61,293	1.00 (0.99–1.01)
Community-level poverty			
Low	878	43,577	Reference
High	1,402	40,805	0.99 (0.98–0.99)*
Community-level maternal literacy			
Low	2,053	51,866	Reference
High	227	32,516	1.05 (1.04–1.06)*
Previous CS delivery			
No	2,239	75,000	Reference
Yes	41	9,325	1.35 (1.33–1.39)*
Type of pregnancy			
Wanted	1,403	73,893	Reference
Unwanted	400	7,371	0.74 (0.72–0.77)*

CS, Cesarean section.

* Significant at *p*-value <0.05.

Controlling for other variables, pregnant women in the age groups of 20–24, 25–29, 30–34, 35–39, and 40–44 years exhibited higher rates of ANC booking, 46% (AHR = 1.46, 95% CI: 1.40–1.53), 40% (AHR = 1.40, 95% CI: 1.35–1.47), 30% (AHR = 1.30, 95% CI: 1.20–1.90), 30% (AHR = 1.30, 95% CI: 1.20–1.34), and 7% (AHR = 1.07, 95% CI: 1.06–1.09), respectively, when compared to those aged 15–19 years. Conversely, pregnant women aged 44–49 had an 8% lower ANC booking initiation rate (AHR = 0.92, 95% CI: 0.90–0.97) compared to those aged 15–19 years.

Keeping other factors constant, ANC booking among pregnant women with a primary education, secondary education, or higher education were 1%, 3%, and 40% higher than those with no education (AHR = 1.01, 95% CI: 1.02–1.25; AHR = 1.03, 95% CI: 1.02–1.05; and AHR = 1.40, 95% CI: 1.30–1.50, respectively).

Among Muslim women, the time to ANC booking was 7% lower (AHR = 0.93, 95% CI: 0.92–0.92) compared to Christian women.

Pregnant women living in a household with a female head had 4% lower rates of ANC booking (AHR = 0.96, 95% CI: 0.95–0.97) compared to those living in a household with a male head. In addition, pregnant women with household heads aged 22–34, 35–64, and over 64 years had higher rates of ANC booking, with increases of 6%, 9%, and 6%, respectively (AHR = 1.06, 95% CI: 1.03–1.08; AHR = 1.09, 95% CI: 1.06–1.10; and AHR = 1.06, 95% CI: 1.02–1.09) when compared to those in the 15–21-year age group.

Pregnant women whose husbands had completed primary, secondary, or higher education had higher ANC booking rates, 8%, 12%, and 8%, respectively (AHR = 1.08, 95% CI: 1.06–1.09; AHR = 1.12, 95% CI: 1.10–1.13, and AHR = 1.08, 95% CI: 1.07–1.10) compared to those whose husbands had no formal education.

Pregnant women living in households with six to seven or more than seven members demonstrated increased rates of ANC bookings of 2% and 7%, respectively (AHR = 1.02, 95% CI: 1.01–1.02 and AHR = 1.07, 95% CI: 1.06–1.08), in comparison with those residing in households with one to five members.

Pregnant women living in households with a middle or rich wealth index had higher rates of ANC booking, 66% and 60% (AHR = 1.66, 95% CI:1.60–1.70 and AHR = 1.60, 95% CI: 1.56–1.73), respectively, compared to those in households with the poor wealth index.

Pregnant women residing in communities with a greater proportion of educated women were 5% more likely to make ANC bookings (AHR = 1.05, 95% CI: 1.04–1.06) compared to those in communities with a lower percentage of educated women.

Pregnant women residing in communities with high poverty were less likely to make an ANC booking (AHR = 0.99, 95% CI: 0.98–0.99) compared to those in communities with a higher wealth index.

Pregnant women with a previous history of CS delivery had a 35% higher rate of ANC booking (AHR = 1.35, 95% CI: 1.33–1.39) compared to those without such a history.

Pregnant women with unwanted pregnancies had a 26% lower rate of ANC booking (AHR = 0.74, 95% CI: 0.72–0.77) compared to those with wanted pregnancies after controlling for other variables (Table 3).

## Discussion

Recent DHS data were utilized to conduct a Weibull gamma shared frailty parametric survival analysis, aiming to ascertain the average time to ANC booking among pregnant women in East African countries and identify the predictors influencing this timing. This study revealed that the median time to ANC booking among pregnant women in East Africa was 4 months, with an interquartile range of 2 months (ranging from 2 to 6 months or 8 to 24 weeks), which is later than the WHO recommendation of 12 weeks (3 months) of gestation (3). A possible reason for this delay compared to the WHO recommendation of 12 weeks (3 months) might be that, in East Africa, there are geographic barriers, such as distance to healthcare facilities, that can significantly impact when women make an ANC booking. In rural areas, women might have to travel long distances to reach a clinic, which can delay their first visit (27, 28).

This evidence was congruent with a previous study’s findings in Ethiopia (18). However, our time to ANC booking was shorter and earlier compared to other previous studies (10, 19, 29, 30). This variation may be attributed to several factors. First, socioeconomic influences play a significant role: women from higher socioeconomic backgrounds often have greater resources to seek care earlier than those with lower socioeconomic status (31). In addition, differences in study design and population characteristics such as variations in sample sizes and methodologies can result in differing findings. Finally, the observed slight improvement in time to ANC booking in East Africa compared to previous studies may be linked to changes in health policies or programs aimed at enhancing maternal health following the WHO recommendations. These initiatives could have facilitated earlier ANC attendance in these regions, reflecting advancements in public health strategies (32, 33).

The study found that factors such as the ages of the women and the household head, the educational status of the mother and her husband, the sex of the household head, household size, wealth index, community-level maternal literacy, community-level poverty, history of previous CS delivery, and type of pregnancy were found to be predictors of the timing of the ANC booking. This finding is supported by the findings of previous studies (11, 16, 18, 34–37).

This study indicates that middle-aged pregnant women and those with older household heads are more likely to schedule their ANC booking earlier. On the other hand, the older age of pregnant women was negatively associated with an early ANC booking. This study finding is similar to the findings of studies conducted in sub-Saharan Africa (38) and Ethiopia (14). This may be because middle-aged pregnant women are more likely to schedule their ANC booking earlier as they often have a better understanding of the importance of early prenatal care (39). On the other hand, older pregnant women may be less likely to initiate early ANC booking due to a sense of confidence from previous pregnancies, leading them to believe that they do not need early medical intervention and the older women might face more physical and logistical challenges, such as mobility issues or other health conditions, which can delay their ability to seek early care (40).

This study indicates that pregnant women who were in households with heads of a higher age were more likely to schedule their ANC booking earlier. This study finding is similar to the findings of studies conducted in sub-Saharan Africa (38) and Ethiopia (14). This may be because older household heads might have more experience and awareness about the importance of a timely ANC booking. Furthermore, older household heads may typically have more decision-making power within the family and are often more financially stable compared to younger ones which makes it easier for them to schedule an ANC booking.

ANC bookings were lower among Muslim women compared to Christian women and this was similar to another study (41). Research on the maternity experiences of English-speaking Muslim women in the United Kingdom indicated that religious beliefs significantly influenced their health-seeking behaviors during pregnancy (42). This may be due to differences in religious practices in some Muslim communities and religious beliefs affecting the seeking of medical care during pregnancy. Therefore, as a study highlighted, healthcare professionals need to be aware of and understand religious values when providing maternity care to Muslim women (43).

This study revealed that the higher educational status of pregnant women and their husbands was positively associated with the time of the ANC booking. This was supported by the findings of other previous studies (18, 29, 36, 44). This may be due to educated husbands having a better ability to convince their spouses to start prenatal care at an early stage of the pregnancy because they have knowledge about maternal and child health (45). Furthermore, educated mothers are more likely to understand the necessities during pregnancy, the importance of ANC, and the benefits of early booking, leading them to schedule their appointments on time.

Time to ANC booking was directly associated with larger households. This is supported by other research findings (18). A possible reason may be that larger households might have more resources, such as financial support, as well as support systems such as more family members available to provide support, encourage timely healthcare visits, and share responsibilities and household duties. Thus, pregnant women might have more time to prioritize their health and attend ANC appointments early.

Time to ANC booking was indirectly associated with a female household head. This may possibly be due to a lack of support from their partner (husband) which may hinder a discussion about utilizing maternal health services (45, 46).

Time to ANC booking was directly associated with the middle and rich levels of household wealth status (29, 36, 47). A possible reason may be that the richest households produce women who are more independent, educated, and confident when seeking maternal health services.

A high proportion of educated mothers in the community was directly associated with the time to ANC booking and a high proportion of poor in the community lowers the initiation of the ANC booking (5, 10, 18, 30). A possible reason could be that educated women are more likely to be aware of the importance of an early ANC booking. They tend to have better access to health information, understand the benefits of early and regular prenatal care, and are more likely to prioritize their health and that of their unborn child. On the other hand, a high proportion of poor individuals in the community lowers the initiation of the first-time ANC booking due to several factors such as financial constraints, lack of transportation, and other socioeconomic barriers.

The initiation of the time to ANC booking was higher among pregnant women who had a previous history of CS delivery. This finding was supported by another study (9). However, this study had no details regarding the size of the disparity between the two groups. It may be due to an increased awareness of pregnancy risks or greater engagement with healthcare services following a CS.

The likelihood of an early ANC booking was higher among pregnant women who wanted to be pregnant compared to pregnant women who had unwanted pregnancies. This finding was similar to the findings of previous studies (9, 11). This might be due to the fact that if a woman wants to become pregnant, they might know they are pregnant and make a timely ANC booking. Furthermore, if they know that they are pregnant early, they might be interested in and care about the pregnancy and make a timely ANC booking.

Regarding the practical and policy implications of this research, the findings indicate that many women in East Africa are attending their first antenatal care visit later than the WHO recommends, particularly among younger and less educated women. Policymakers should focus on developing healthcare policies that prioritize early antenatal care initiation by promoting maternal education and enhancing economic conditions to support timely access to care.

The strength of this study is the use of nationally representative data, which allows it to be generalizable to all pregnant women in East African countries. The DHS surveys rely on self-reported information, which can be subject to recall bias or social desirability bias, and this may be a limitation of this study.

## Conclusion

The median time of an ANC booking among pregnant women in East African countries is later than the new WHO recommendation for ANC booking for a positive pregnancy experience. The study found that a woman being middle-aged, having an older household head, a higher maternal and husband education level, higher community-level maternal literacy, a higher wealth index, and having previous CS delivery increased the likelihood of an early ANC booking. Conversely, a woman being of an older age, a female household head, and having an unwanted pregnancy lowered the likelihood of an early ANC booking. Therefore, strengthening systematic efforts to improve the educational status of women and their husbands, encouraging women's education in the community, providing economic support for women with a low-wealth status and poor communities, encouraging wanted pregnancies, and providing accessible counseling services for women who have unwanted pregnancies will help to encourage early ANC booking among pregnant women in East Africa.

## Data Availability

The original contributions presented in the study are included in the article/Supplementary Material, further inquiries can be directed to the corresponding author.

## References

[1] World Health Organization. WHO Recommendations on Interventions to Improve Preterm Birth Outcomes. Geneva: World Health Organization (2015). 26447264

[2] World Health Organization. WHO Recommendations on Maternal Health: Guidelines Approved by the WHO Guidelines Review Committee. Geneva: World Health Organization (2017). 40455892

[3] WHO. New Guidelines on Antenatal Care for a Positive Pregnancy Experience. Geneva: World Health Organization (2016). 28079998

[4] WHO. Maternal Mortality Fact Sheet. Geneva: World Health Organization (2022).

[5] Group WB. Trends in Maternal Mortality: 2000–2017. Geneva: World Health Organization (2017).

[6] Boldosser-Boesch A Brun M Carvajal L Chou D de Bernis L Fogg K Setting maternal mortality targets for the SDGs. Lancet. (2017) 389(10070):696–7. 10.1016/S0140-6736(17)30337-9 28229871

[7] Afshin A Sur PJ Fay KA Cornaby L Ferrara G Salama JS Health effects of dietary risks in 195 countries, 1990–2017: a systematic analysis for the global burden of disease study 2017. Lancet. (2019) 393(10184):1958–72. 10.1016/S0140-6736(19)30041-8 30954305 PMC6899507

[8] Alkema L Chou D Hogan D Zhang S Moller AB Gemmill A Global, regional, and national levels and trends in maternal mortality between 1990 and 2015, with scenario-based projections to 2030: a systematic analysis by the UN maternal mortality estimation inter-agency group. Lancet. (2016) 387(10017):462–74. 10.1016/S0140-6736(15)00838-7 26584737 PMC5515236

[9] Gebresilassie B Belete T Tilahun W Berhane B Gebresilassie S. Timing of first antenatal care attendance and associated factors among pregnant women in public health institutions of Axum town, Tigray, Ethiopia, 2017: a mixed design study. BMC Pregnancy Childbirth. (2019) 19:1–11. 10.1186/s12884-019-2490-5 31533657 PMC6751589

[10] Fentaw KD Fenta SM Biresaw HB Mulugeta SS. Time to first antenatal care visit among pregnant women in Ethiopia: secondary analysis of EDHS 2016; application of AFT shared frailty models. Arch Public Health. (2021) 79:1–14. 10.1186/s13690-021-00720-2 34749787 PMC8576895

[11] Zegeye AM Bitew BD Koye DN. Prevalence and determinants of early antenatal care visit among pregnant women attending antenatal care in Debre Berhan health institutions, central Ethiopia. Afr J Reprod Health. (2013) 17(4):4–5. 24558789

[12] Ng’Ambi WF Collins JH Colbourn T Mangal T Phillips A Kachale F Socio-demographic factors associated with early antenatal care visits among pregnant women in Malawi: 2004–2016. PLoS One. (2022) 17(2):e0263650 10.1371/journal.pone.0263650 35134088 PMC8824333

[13] Ewunetie AA Munea AM Meselu BT Simeneh MM Meteku BT. DELAY on first antenatal care visit and its associated factors among pregnant women in public health facilities of Debre Markos town, north west Ethiopia. BMC Pregnancy Childbirth. (2018) 18:1–8. 10.1186/s12884-018-1748-7 29769122 PMC5956942

[14] Redi T Seid O Bazie GW Amsalu ET Cherie N Yalew M. Timely initiation of antenatal care and associated factors among pregnant women attending antenatal care in southwest Ethiopia. PLoS One. (2022) 17(8):e0273152. 10.1371/journal.pone.0273152 35980904 PMC9387795

[15] Ekholuenetale M Benebo FO Idebolo AF. Individual-, household-, and community-level factors associated with eight or more antenatal care contacts in Nigeria: evidence from Demographic and Health Survey. PLoS One. (2020) 15(9):e0239855. 10.1371/journal.pone.0239855 32976494 PMC7518602

[16] Belay A Astatkie T Abebaw S Gebreamanule B Enbeyle W. Prevalence and factors affecting the utilization of antenatal care in rural areas of southwestern Ethiopia. BMC Pregnancy Childbirth. (2022) 22(1):30. 10.1186/s12884-021-04362-8 35031008 PMC8759251

[17] Tessema ZT Tesema GA Yazachew L. Individual-level and community-level factors associated with eight or more antenatal care contacts in sub-Saharan Africa: evidence from 36 sub-Saharan African countries. BMJ Open. (2022) 12(3):e049379. 10.1136/bmjopen-2021-049379 35273040 PMC8915341

[18] Kitaw TA Haile RN. Time to first antenatal care booking and its determinants among pregnant women in Ethiopia: survival analysis of recent evidence from EDHS 2019. BMC Pregnancy Childbirth. (2022) 22(1):921. 10.1186/s12884-022-05270-1 36482385 PMC9733146

[19] Dewau R Muche A Fentaw Z Yalew M Bitew G Amsalu E Time to initiation of antenatal care and its predictors among pregnant women in Ethiopia: Cox-gamma shared frailty model. PLoS One. (2021) 16(2):e0246349. 10.1371/journal.pone.0246349 33544714 PMC7864666

[20] Adewuyi EO Auta A Khanal V Bamidele OD Akuoko CP Adefemi K Prevalence and factors associated with underutilization of antenatal care services in Nigeria: a comparative study of rural and urban residences based on the 2013 Nigeria Demographic and Health Survey. PLoS One. (2018) 13(5):e0197324. 10.1371/journal.pone.0197324 29782511 PMC5962076

[21] Mugo NS Dibley MJ Agho KE. Prevalence and risk factors for non-use of antenatal care visits: analysis of the 2010 South Sudan household survey. BMC Pregnancy Childbirth. (2015) 15:1–13. 10.1186/s12884-015-0429-z 25885187 PMC4396873

[22] Tsegaye B Ayalew M. Prevalence and factors associated with antenatal care utilization in Ethiopia: an evidence from demographic health survey 2016. BMC Pregnancy Childbirth. (2020) 20:1–9. 10.1186/s12884-020-03236-9 PMC7488553 32917156

[23] Belay DG Alemu MB Aragaw FM Asratie MH. Time to initiation of antenatal care visit and its predictors among reproductive age women in Ethiopia: Gompertz inverse Gaussian shared frailty model. Front Glob Womens Health. (2023) 4:917895. 10.3389/fgwh.2023.917895 37854167 PMC10579888

[24] Statistics D. Guide-to-DHS-Statistics. Organization of DHS Data.

[25] Daniel J Corsi MN Finlay JE Subramanian SV. Demographic and health surveys: a profile. Int J Epidemiol. (2012) 41(6):1602. 10.1093/ije/dys184 23148108

[26] Gutierrez R. Parametric frailty and shared frailty survival models. Stata J. (2002) 2(1):22–44. 10.1177/1536867X0200200102

[27] Tegegne TK Chojenta C Loxton D Smith R Kibret KT. The impact of geographic access on institutional delivery care use in low and middle-income countries: systematic review and meta-analysis. PLoS One. (2018) 13(8):e0203130. 10.1371/journal.pone.0203130 30161201 PMC6117044

[28] Wigley A Tejedor-Garavito N Alegana V Carioli A Ruktanonchai CW Pezzulo C Measuring the availability and geographical accessibility of maternal health services across sub-Saharan Africa. BMC Med. (2020) 18:1–10. 10.1186/s12916-020-01707-6 32895051 PMC7487649

[29] Kisuule I Kaye DK Najjuka F Ssematimba SK Arinda A Nakitende G Timing and reasons for coming late for the first antenatal care visit by pregnant women at Mulago hospital, Kampala Uganda. BMC Pregnancy Childbirth. (2013) 13:1–7. 10.1186/1471-2393-13-121 23706142 PMC3665546

[30] Seid A Ahmed M. Survival time to first antenatal care visit and its predictors among women in Ethiopia. PLoS One. (2021) 16(5):e0251322. 10.1371/journal.pone.0251322 33956902 PMC8101713

[31] Tikmani SS Ali SA Saleem S Bann CM Mwenechanya M Carlo WA Trends of antenatal care during pregnancy in low-and middle-income countries: findings from the global network maternal and newborn health registry. Semin Perinatol. (2019) 43(5):297–307. 10.1053/j.semperi.2019.03.020 31005357 PMC7027164

[32] Mchenga M Burger R Von Fintel D. Examining the impact of WHO’s focused antenatal care policy on early access, underutilisation and quality of antenatal care services in Malawi: a retrospective study. BMC Health Serv Res. (2019) 19:1–14. 10.1186/s12913-019-4130-1 31068183 PMC6506931

[33] Habte A Tamene A Melis T. Compliance towards WHO recommendations on antenatal care for a positive pregnancy experience: timeliness and adequacy of antenatal care visit in sub-Saharan African countries: evidence from the most recent standard demographic health survey data. PLoS One. (2024) 19(1):e0294981. 10.1371/journal.pone.0294981 38271342 PMC10810464

[34] Seyoum K Tekalegn Y Quisido B. Determinants and prevalence of early initiation of breastfeeding: does the place of delivery matter? A comparative cross-sectional study based on the 2016 Ethiopian Demographic and Health Survey data. Popul Med. (2021) 3(December):1–8. 10.18332/popmed/144318

[35] Adulo LA Hassen SS. Magnitude and factors associated with late initiation of antenatal care booking on first visit among women in rural parts of Ethiopia. J Racial Ethn Health Disparities. (2023) 10(4):1693–702. 35761146 10.1007/s40615-022-01354-y

[36] Halima SE-S Eid SM El-Bassioune WM. Prevalence and determinants of first antenatal care visit among pregnant women attending public health institutions at Damietta governorate, Egypt. Int J Med Arts. (2021) 3(2):1215–20.

[37] Alamneh A Asmamaw A Woldemariam M Yenew C Atikilt G Andualem M Trend change in delayed first antenatal care visit among reproductive-aged women in Ethiopia: multivariate decomposition analysis. Reprod Health. (2022) 19(1):80. 10.1186/s12978-022-01373-2 35346248 PMC8962488

[38] Alem AZ Yeshaw Y Liyew AM Tesema GA Alamneh TS Worku MG Timely initiation of antenatal care and its associated factors among pregnant women in sub-Saharan Africa: a multicountry analysis of demographic and health surveys. PLoS One. (2022) 17(1):e0262411 10.1371/journal.pone.0262411 35007296 PMC8746770

[39] Fantaye AW Okonofua F Ntoimo L Yaya S. A qualitative study of community elders’ perceptions about the underutilization of formal maternal care and maternal death in rural Nigeria. Reprod Health. (2019) 16:1–17. 10.1186/s12978-019-0831-5 31711527 PMC6849176

[40] Appiah F. Individual and community-level factors associated with early initiation of prenatal care: multilevel modeling of 2018 Cameroon Demographic and Health Survey. PLoS One. (2022) 17(4):e0266594. 10.1371/journal.pone.0266594 35385559 PMC8986322

[41] Shiferaw K Mengistie B Gobena T Dheresa M Seme A. Adequacy and timeliness of antenatal care visits among Ethiopian women: a community-based panel study. BMJ Open. (2021) 11(12):e053357. 10.1136/bmjopen-2021-053357 34949623 PMC8704979

[42] Hassan SM Leavey C Rooney JS. Exploring English speaking Muslim women’s first-time maternity experiences: a qualitative longitudinal interview study. BMC Pregnancy Childbirth. (2019) 19:1–10. 10.1186/s12884-019-2302-y 31060520 PMC6501380

[43] Hassan SM. Religious practices of Muslim women in the UK during maternity: evidence-based professional practice recommendations. BMC Pregnancy Childbirth. (2022) 22(1):335. 10.1186/s12884-022-04664-5 35440069 PMC9020041

[44] Abebe GF Alie M Girma D Mankelkl G Berchedi AA Negesse Y. Determinants of early initiation of first antenatal care visit in Ethiopia based on the 2019 Ethiopia mini-demographic and health survey: a multilevel analysis. PLoS One. (2023) 18(3):e0281038. 10.1371/journal.pone.0281038 36877686 PMC9987803

[45] Suandi D Williams P Bhattacharya S. Does involving male partners in antenatal care improve healthcare utilisation? Systematic review and meta-analysis of the published literature from low- and middle-income countries. Int Health. (2020) 12(5):484–98. 10.1093/inthealth/ihz073 31613327 PMC11701106

[46] Kyei-Nimakoh M Carolan-Olah M McCann TV. Access barriers to obstetric care at health facilities in Sub-Saharan Africa—a systematic review. Syst Rev. (2017) 6:1–6. 10.1186/s13643-017-0503-x 28587676 PMC5461715

[47] Jassey B. Prevalence and determinants of early antenatal care visits among pregnant women attending antenatal care in rural Gambia. JMMR. (2023) 12(1):75–87. 10.18196/jmmr.v12i1.32

